# Effect of ZnO Nanofiller on Structural and Electrochemical Performance Improvement of Solid Polymer Electrolytes Based on Polyvinyl Alcohol–Cellulose Acetate–Potassium Carbonate Composites

**DOI:** 10.3390/molecules27175528

**Published:** 2022-08-28

**Authors:** John Ojur Dennis, Mohammed Khalil Mohammed Ali, Khalid Hassan Ibnaouf, Osama Aldaghri, Naglaa F. M. Abdel All, Abdullahi Abbas Adam, Fahad Usman, Yarima Mudassir Hassan, Bashir Abubakar Abdulkadir

**Affiliations:** 1 Department of Fundamental and Applied Sciences, Universiti Teknologi PETRONAS, Seri Iskandar 32610, Malaysia; 2 Department of Physics, College of Science, Imam Mohammad Ibn Saud Islamic University (IMSIU), Riyadh 13318, Saudi Arabia; 3 Department of Physics, Al-Qalam University, Katsina 820102, Nigeria; 4 Department of Chemistry, Gombe State University, Gombe 760253, Nigeria

**Keywords:** cellulose acetate, conductivity, K_2_CO_3_, solid polymer electrolyte, polyvinyl alcohol, zinc oxide

## Abstract

In this study, a solution casting method was used to prepare solid polymer electrolytes (SPEs) based on a polymer blend comprising polyvinyl alcohol (PVA), cellulose acetate (CA), and potassium carbonate (K_2_CO_3_) as a conducting salt, and zinc oxide nanoparticles (ZnO-NPs) as a nanofiller. The prepared electrolytes were physicochemically and electrochemically characterized, and their semi-crystalline nature was established using XRD and FESEM. The addition of ZnO to the polymer–salt combination resulted in a substantial increase in ionic conductivity, which was investigated using impedance analysis. The size of the semicircles in the Cole–Cole plots shrank as the amount of nanofiller increased, showing a decrease in bulk resistance that might be ascribed to an increase in ions due to the strong action of the ZnO-NPs. The sample with 10 wt % ZnO-NPs was found to produce the highest ionic conductivity, potential window, and lowest activation energy (*E_a_*) of 3.70 × 10^–3^ Scm^–1^, 3.24 V, and 6.08 × 10^–4^ eV, respectively. The temperature–frequency dependence of conductivity was found to approximately follow the Arrhenius model, which established that the electrolytes in this study are thermally activated. Hence, it can be concluded that, based on the improved conductivity observed, SPEs based on a PVA-CA-K_2_CO_3_/ZnO-NPs composite could be applicable in all-solid-state energy storage devices.

## 1. Introduction

Electrolytes have been recognized as one of the main components of energy storage devices such as fuel cells, batteries, and, recently, supercapacitors. This is because ion movement and conduction mechanisms occur exclusively within the electrolyte materials. Electrolytes in energy storage devices have an important role in establishing critical properties in numerous areas, such as determining the role of voltage in the energy density of the electrochemical supercapacitors, internal resistance, cycling lifetime, power density, temperature range, rate performance, and self-discharge, which are all important in the practical application of supercapacitors [1,2]. The ionic conductivity of electrolytes plays an important role in the internal resistance of supercapacitors [2]. Electrolytes fill the gap between the electrodes, allowing ions to pass through. It is a crucial component in the creation of energy storage since the maximal working voltage is determined by the electrolyte’s breakdown voltage [3]. Electrolytes also affect the equivalent series resistance (ESR), which is a determinant factor of the power density. Electrolytes are normally acids, bases, or salts in solution or solid form, and some have been reported to exist as gas electrolytes under certain conditions, such as high temperature and low pressure [4]. Liquid electrolytes have better conductivity than solid electrolytes [5,6]. However, they have a number of limitations, such as environmental unfriendliness, leakage issues, limited cycle life, long charging time, and a low potential window [7,8]. Therefore, improvement in energy storage devices depends profoundly on the development of new types of electrolytes called solid polymer electrolytes (SPEs) [9]. 

Recent research on electrolytes, particularly SPEs, is generally driven by the safety of the electrolytes and their good electrochemical performance [10]. SPEs promise better performance for lithium batteries, sensors, fuel cells, supercapacitors, and other devices [11]. SPEs exhibit realistic potential window stability, are easy to make, and have high thermal stability, according to previous studies [12]. Different efforts were made to increase the ionic conductivity of SPEs, including polymer blends and the addition of fillers [13,14,15,16]. Blending polymers is a suitable technique to obtain a good mix of physical qualities and superior attributes, and the miscibility of the components is the most significant element to consider when choosing polymer blends [17]. Accordingly, there is increasing interest throughout the scientific community in making SPE blends based on “green or natural polymers” typically obtained from renewable and natural sources, owing to their environmental friendliness. Due to the renewable and biodegradable nature of biopolymers, different studies were conducted to replace the current fossil fuel with natural polymers. The use of different biopolymers was reported earlier; however, in order to further enhance the performance and other important properties, such as the thermal and mechanical properties of SPEs based on biopolymers, it is vital to blend two natural polymers together. It was reported that the polymer blending technique is useful in the improvement of the electrochemical performance of SPEs [5]. Polymers such as cellulose acetate (CA) and polyvinyl alcohol (PVA) were reported to exhibit some potential advantages due to their ease of production [18,19,20,21]. The existence of polar groups in CA makes it an appropriate material to blend with other polymers, such as PVA [22]. Hence, the synthesis of SPEs based on polymer blends is proven to enhance conductivity of an electrolyte due to the suitable morphological and structural properties of the two polymers (PVA and CA). 

However, fundamental issues such as low conductivity and poor cycle stability must be addressed before PVA–CA can be used as SPE material with improved performance. The modification of polymers is required to improve the performance of SPEs based on blended PVA–CA. The incorporation of salt into a polymer is a promising technique for achieving synergistic electrochemical results. From both fundamental and application standpoints, incorporating salt into the blended polymer matrix is a focus of research nowadays [17,23]. Various conducting salts are added to enhance the characteristics of the polymers for SPEs. Additives such as transition metals have been added that have a considerable influence on optical and electrical properties. Different studies were reported using different salts to enhance the characteristics of polymer blends when combined with acids or salts. [12,14]. Kadir et al., for instance, reported the influence of salt on the performance of the PVA and chitosan for SPE [21]. The study established that the performance of PVA and chitosan at ambient temperature is insignificant. Nevertheless, an improvement in the performance of the electrolyte was observed after successful incorporation of ethylene carbonate (EC) and NH_4_NO_3_. 

Consequently, to further improve ionic conductivity, chemical stability, and other important electrochemical performances of SPEs, the use of different active fillers is employed [24]. The inclusion of fillers is said to be one of the common ways to improve ion mobility and interfacial contact between ions and polar groups within the electrolytes [16,25]. Ion transport is further hindered by the crystalline structure of certain polymers in SPEs [26]. Blending the polymer with an appropriate filler is found to be the most active strategy to lessen the crystallinity of the polymer and improve conductivity. Furthermore, the addition of filler can change the electrolyte’s polymeric composition and improve its conductivity. Film absorptivity and mechanical qualities have been observed to be influenced by the filler chosen and its concentration [16,27].

Zinc oxide nanoparticles (ZnO-NPs) are wide-bandgap semi-conductors that have opto-electrical features. Many SPE systems employ ZnO-NPs to enhance the conductivity, structural characteristics, and other properties of the composite SPEs generated [28]. For instance, Zebardastan et al. [29] reported the performances of SPEs involving various amounts of ZnO in the PVdF-HFP:PEO:EC:PC:NaI:I_2_:ZnO, where the maximum conductivity was achieved with 3 wt % ZnO nano filler. The increase in conductivity was attributed by the authors to an increase in the amorphous portion of the electrolyte. Similarly, the effect of ZnO-NPs on the mechanical and thermal properties of PVA was reported, with the findings indicating that adding ZnO-NPs improved the attributes of PVA [28]. Recently, a study on proton-conducting composite SPEs based on PVA/NH_4_NO_3_ reported that the incorporation of small amounts of ZnO-NPs considerably improved the proton conduction of the electrolyte [30]. Nevertheless, in all the electrolytes reported earlier using ZnO nanofiller, the conductivity was found to be low, and thus, further improvement is necessary. 

Currently, the study of ZnO as a filler in polymer blend SPEs comprising PVA–CA–K_2_CO_3_ with the aim to further improve conductivity is yet to be reported in the literature. Based on the optimization study conducted as reported in our previous study, SPEs based on a PVA–CA blend with 20 wt % of K_2_CO_3_ display the highest amorphous structure and a reasonable conductivity of 5.30 × 10^−4^ Scm^−1^ [31]. Although the conductivity of this SPE is improved by the addition of K_2_CO_3_, it is still inadequate for practical applications. Accordingly, in this article, the effects of ZnO-NPs on the electrochemical performance of SPEs based on the optimized PVA–CA with incorporated 20% K_2_CO_3_ are investigated. The electrolytes were prepared through a solution cast procedure and the developed SPEs were characterized physicochemically and electrochemically.

## 2. Materials and Methods

### 2.1. Materials

PVA and CA (hydrolyzed 99%), dimethyl sulfoxide (DMSO), ZnO-NPs (~10 nm), and potassium carbonate (K_2_CO_3_) (anhydrous) were purchased from Sigma-Aldrich through Avantis Chemicals Supply (Ipoh-Perak, Malaysia), and all the reagents were used as received. PVA and CA were used as polymers, DMSO as a solvent, K_2_CO_3_ as a salt, and ZnO-NPs as nanofiller.

### 2.2. Synthesis of PVA–CA–K_2_CO_3_/ZnO-NPs Composite

**Figure 1:**
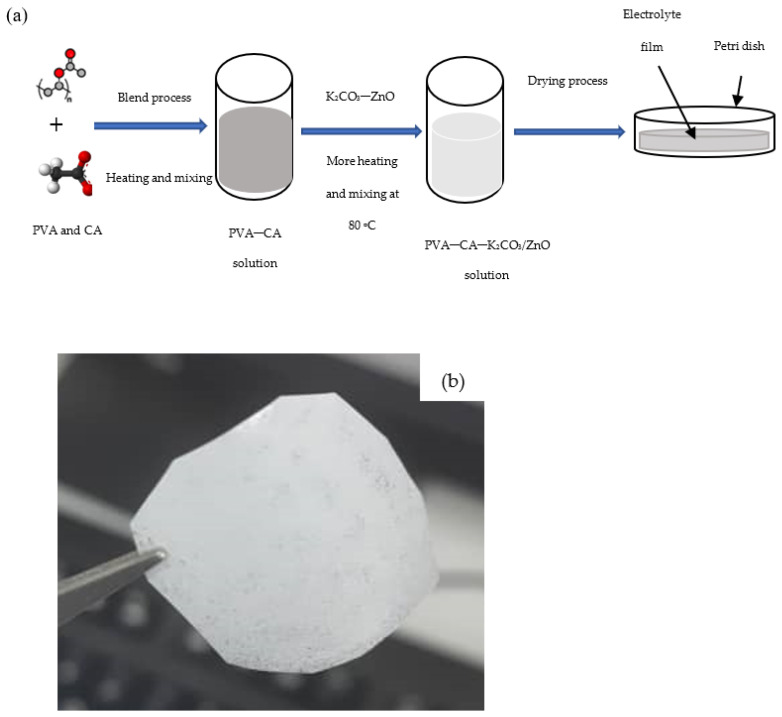
(**a**) Illustration steps for the preparation of SPEs, and (**b**) typical photo of the PVA–CA–K_2_CO_3_/ZnO-NPs SPE film with the composition of 10 wt % ZnO-NPs.

A detailed description of how the optimum PVA–CA–K_2_CO_3_ composite SPEs were prepared is given in our previous work [31]. To prepare the nanofiller containing SPEs, different wt % of ZnO-NPs were incorporated into the optimized PVA–CA–K_2_CO_3_ SPE, and the mixtures were heated until a clear solution was obtained. The resultant solution was transferred into Petri dishes for drying purposes. The thickness of the dry samples was measured using a digital micrometer screw gauge. Figure 1a shows an illustration of the preparation steps and Figure 1b shows a photo of a typical PVA–CA–K_2_CO_3_/ZnO-NPs film with the composition of 10 wt % ZnO-NPs (10 wt %).

To protect the samples from any trace of moisture, the prepared SPE membranes were preserved in a desiccator before characterization. To obtain the optimum composition of ZnO-NPs, the quantity of ZnO-NPs was varied while keeping the PVA–CA–K_2_CO_3_ fraction constant, as presented in Table 1. The resultant samples were coded as PZ0, PZ5, PZ10, PZ15, and PZ20 for samples incorporated with 0, 5, 10, 15, and 20 wt % ZnO-NPs, respectively. 

### 2.3. Physicochemical Characterization

X-ray diffraction (XRD) was utilized to examine the crystal phase of the samples and it was carried out using a Bruker D8 Advance Power diffractometer with Cu K radiation (λ = 1.54) for a 2-scattering angle (0° to 100°) at a rate of 5°/min. Fourier-transform infrared spectroscopy (FTIR) using PerkinElmer Spectrum One (Bruker Instruments, Oberkochen, Germany) at a wavenumber of 500 to 4000 cm^−1^ with resolution of 4 cm^−1^ was utilized to study the interaction and coordination between the polymers and the salt. Field emission scanning electron microscopy/energy-dispersive X-ray (FESEM/EDX) with a Zeiss Supra 55 VP scanning electron microscope (Oberkochen, Germany) with a magnification scale of 500 k was used to analyze the morphology and elemental composition of the electrolytes [32]. Thermal properties of the electrolytes were investigated by means of differential scanning calorimetry (DSC) (Model DSC Q2000 V24.11, Oberkochen, Germany). The samples’ glass transition temperatures (*T_g_*) were measured at a scan rate of 10 °C/m from 50 °C to 400 °C in a N_2_ environment. Under nitrogen flow, a sample weight of roughly 4 mg was employed.

### 2.4. Electrochemical Characterization

Electrochemical impedance spectroscopy (EIS) was carried out with a Metrohm Multi Autolab M204 in the frequency range of 10 Hz to 10^5^ Hz. The ionic conductivity of the electrolyte was deduced from EIS study. Subsequently, using a two-electrode system, linear sweep voltammetry (LSV) was utilized to examine the electrochemical stability of the sample. The stability range was deduced using an AU-TOLAB/AUT51018 electrochemical analyzer at a scan rate of 10 mVs^−1^ [16].

## 3. Results

### 3.1. Physicochemical Characterization 

#### 3.1.1. Crystal Phase for PVA–CA–K_2_CO_3_/ZnO-NPs

**Figure 2:**
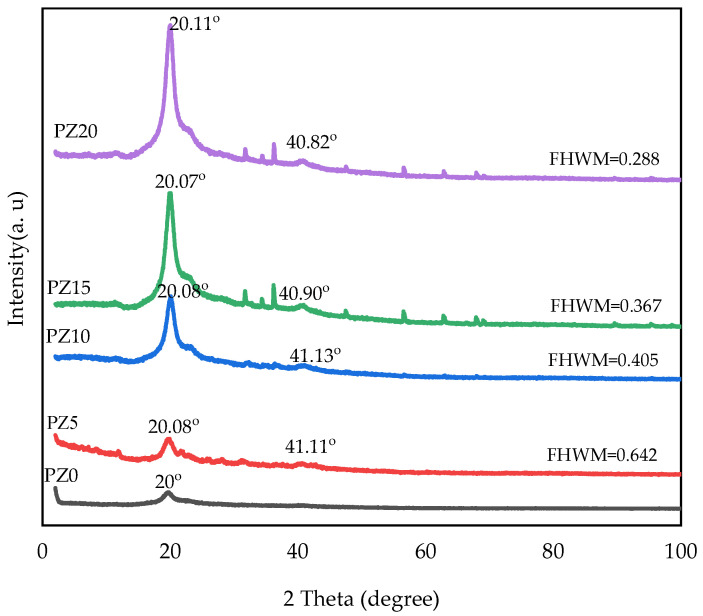
XRD pattern of PVA–CA–K_2_CO_3_ composite SPEs (PZ0) and PVA–CA–K_2_CO_3_ composite SPEs with varying concentrations of ZnO-NPs from 5 to 20 wt % (PZ5 to PZ20).

In our previous study, we reported the XRD peaks of pristine PVA, CA, and PVA–CA–K_2_CO_3_ composites [31]. Accordingly, we determined that 20 wt % K_2_CO_3_ resulted in the most amorphous sample in the group. Therefore, further study with the addition of nanofillers and testing and characterization at different conditions were based on this optimized combination [31]. Figure 2 displays the diffraction pattern of the electrolytes for the PVA–CA–K_2_CO_3_ SPE with no nanofiller (PZ0) and SPE samples of PVA–CA–K_2_CO_3_ containing varying concentrations in wt % of ZnO nanofiller. The XRD arrays for all electrolytes displayed a broad peak at 2θ = 19.25°, which corresponds to the semi-crystalline structure of the parent PVA arising from the intra- and inter-molecular connection of hydrogen of the OH group in the backbone of PVA. It could be observed that the regular structural framework of PVA was conserved even after the incorporation of ZnO-NPs, which could be attributed to the adequate distribution of the nanofiller into the PVA–CA–K_2_CO_3_ matrix resulting in the successful formation of PVA–CA–K_2_CO_3_/ZnO-NPs SPE composites [30].

As can be observed, the lower peak intensity characteristic at about 2θ = 41° is exhibited upon loading 5–10 wt % ZnO-NPs, indicating the more amorphous nature of the samples, and this may result in a major conductivity increase in PVA–CA–K_2_CO_3_/ZnO-NPs SPEs. Similarly, the XRD patterns show that the main diffraction peak of PZ5 and PZ10 at 2θ = 20° is weaker than the other samples (PZ15-PZ20). Furthermore, PZ5 and PZ10 show a larger full width at half maximum (FWHM) value and a lower peak intensity, which suggests that an appropriate quantity of ZnO-NPs could reduce the crystalline phase of PVA–CA–K_2_CO_3_, thus increasing the sample’s ionic conductivity. The low peak intensity observed could be due to a further drop in PVA crystallinity with the addition of ZnO, which is caused by the separation of polymer chains and the reconfiguration of its structure [27]. According to a previous study, the XRD pattern’s low peak intensity indicates a rise in the amorphous region of the electrolytes. As a result of the strong plasticizing impact of the ZnO-NPs, it can be established that adding filler, particularly ZnO-NPs, to the polymer mix matrix can extend the amorphous area [16]. However, beyond the concentration of 10 wt %, the ZnO-NP peaks increase further, with the appearance of new peaks indicating an increase in the crystalline phase of the electrolytes, and subsequently, a decrease in conductivity owing to the re-combination of the dissociated ions. It was stated that any increase in the crystalline phase of the polymers has an adverse effect on the ionic conductivity [30].

#### 3.1.2. FTIR Analysis of PVA–CA–K_2_CO_3_/ZnO-NPs

**Figure 3:**
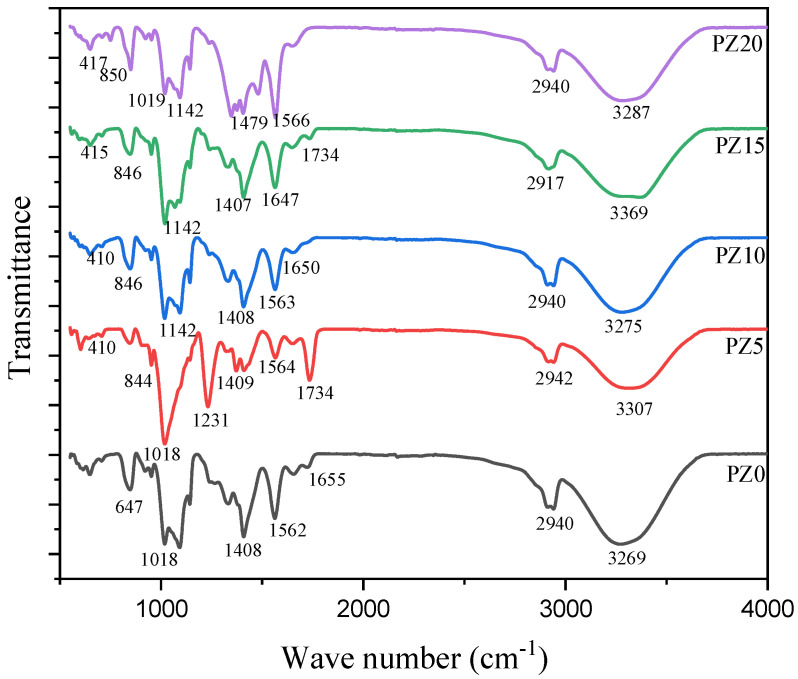
FTIR spectra of pure PVA–CA–K_2_CO_3_ composite SPEs (PZ0) and PVA–CA–K_2_CO_3_ composite SPEs with varying concentrations of ZnO-NPs from 5 to 20 wt % (PZ5 to PZ20).

FTIR analysis was used to study the complex formation between polymers, salt, and the nanofiller. Figure 3 represents the FTIR results of the prepared composites with different nanofiller concentrations in the wavenumber range of 400 to 4000 cm^–1^. The broadening OH from blended polymers is attributed to the peak at 3454 cm^–1^. The band at 2917–2942 cm^–1^ is ascribed to the asymmetric broadening of CH, where the band at 1734 cm^−1^ is credited to carboxyl group (CO) broadening [26,33]. Likewise, the band at 1562–1566 cm^–1^ is attributed to the composite C=C widening and shaking at the α, β-unsaturated ketone. Equally, the 1408 cm^–1^ peak matches CH_2_ twisting vibrations, whereas the peak at 1231 cm^–1^ corresponds to CH widening ambiances and CO widening of the ester group from the CA. The CO enlargement of the acetyl group presence in the PVA and the C–O–C broadening of a pyrose loop in the CA was allocated to the 1019 cm^–1^ band, while the absorption bands at 647 cm^–1^ were allocated to C–H stunning vibration [34,35].

Correspondingly, when K_2_CO_3_ salt and ZnO nanofiller were added to the polymer blends, it could be observed that the strength, location, and form of the peaks differ, signifying a reaction process linking oxygen from CA and the cations from the ZnO [36]. With the incorporation of the ZnO, the wide band at 3454 cm^–1^ that was assigned to the widening and shuddering of OH of the PZ0 altered and extended. All the electrolyte samples (PZ5–PZ20) showed an increase in the OH peaks. The improved intensity of the C=O widening and instability in the bands confirmed that PVA–CA–K_2_CO_3_/ZnO-NPs were effectively formed. This reveals that the Zn^+^ of the nanofiller and the OH of the polymers are matched, where acid–base interactions between the ZnO-NPs and polymer matrix were formed [37,38]. Consequently, to establish the complex formation between polymers, salts, and the nanofiller, variations in band strengths are vital [23]. Similarly, an absorption band located at 410–417 cm^−1^ was linked to the widening bond of Zn-O [39]. The presence of new bands confirmed that the content of ZnO-NPs incorporated into the polymer affected the nature of the peaks, as can be seen in wave numbers 3269 and 3287 cm^–1^. Nevertheless, there are no significant changes in the electrolyte’s wavenumber with the incorporation of ZnO-NPs, which indicates the absence of chemical interactions arising following the incorporation of ZnO-NPs; rather, simply physical interactions between the polymer blend and ZnO nanofiller occurred. Similar findings where no such interactions occur between polymers and ZnO-NPs were reported earlier [27,40].

#### 3.1.3. Morphological and Structural Analysis of PVA–CA–K_2_CO_3_/ZnO-NPs

**Figure 4:**
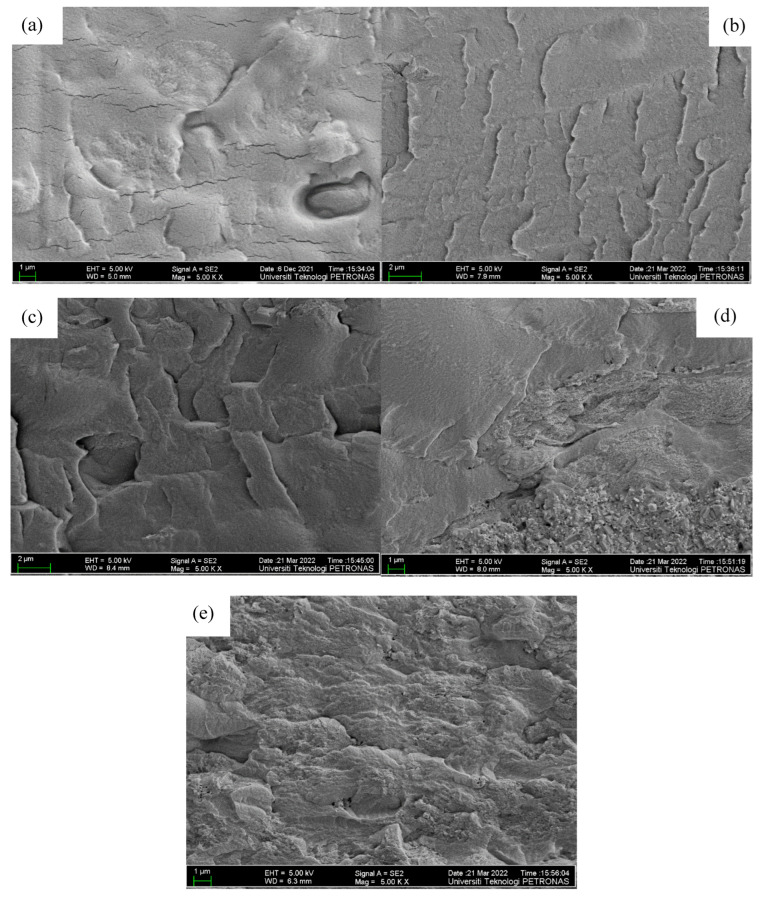
FESEM micrographs of the surface of (**a**) PZ0 (**b**) PZ5 (**c**) PZ10 (**d**) PZ15, and (**e**) PZ20 blend SPEs.

**Figure 5:**
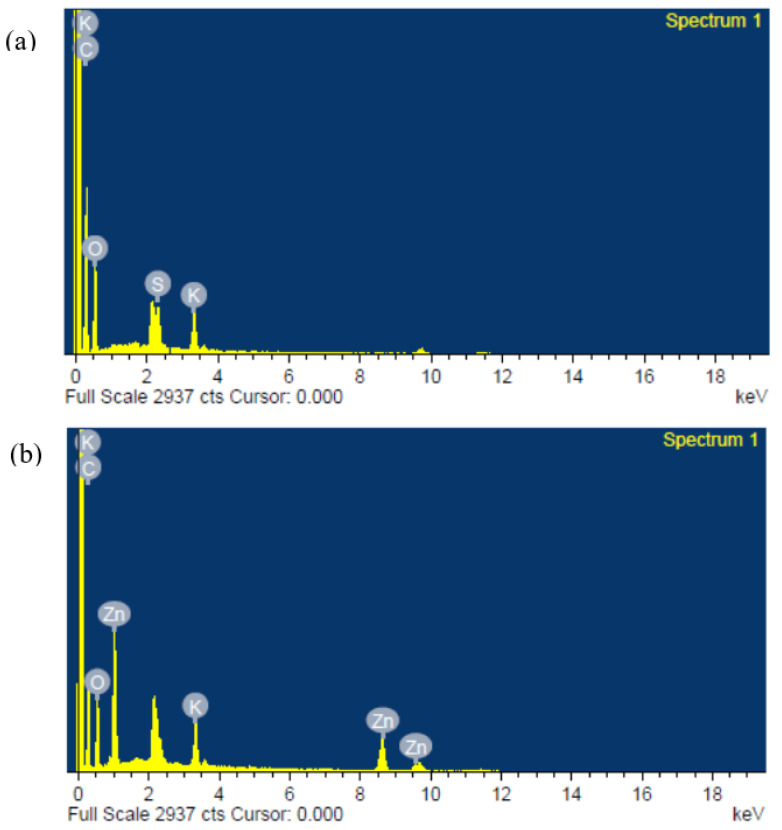
Typical energy-dispersive X-ray (EDX) plots of the composite polymer electrolytes of (**a**) PZ0 and (**b**) PZ10, showing successful incorporation of Zn into PVA–CA–K_2_CO_3_.

Figure 4 shows FESEM micrographs of the external morphology of the polymer blend. The morphology of the sample without nanofiller as shown in Figure 4a is homogeneous and smooth, demonstrating the homogeneousness of the host polymers and the salt, and this is due to the formation of a complex as well as small connections between the OH group of the PVA–CA blend and K^+^ from the salts [16,18]. With the incorporation of the nanofiller, however, it can be noticed that the surface of the sample becomes rough, with bricklike structures, revealing that the ZnO-NPs have been successfully incorporated, and this is corroborated by the elemental peaks in the EDX plots shown in Figure 5. Moreover, with the addition of 5–10 wt % ZnO nanofiller into the electrolyte film, it can be observed that the morphological structure of the samples resulted in a rougher surface morphology, as shown in Figure 4b,c. This designates an improvement in the amorphous region and plasticity of the polymer chain that helps improve the conductivity of the prepared samples. Furthermore, the roughness of the surface morphology of the electrolytes may be ascribed to the decrease in the crystallinity of the PVA–CA–K_2_CO_3_ electrolyte and an increase in the segmental motion of the polymer with the incorporation of ZnO nanofiller, as reported in a previous study [41].

However, further increases in the ZnO filler concentration beyond 10 wt % caused extra accumulation of ZnO particles on the surface of the films, as presented in Figure 4d,e, which might cause a decrease in the conductivity of the film [41]. It was described that excess quantity of filler could result in an irregular distribution and accumulation on the surface polymer hosts, and this could be due to the surface energy and low adhesion within the polymer hosts and ZnO [42]. Therefore, extra agglomerations were detected in the samples with higher wt % ZnO, which could result in the damage of a substantial quantity of ions that could consequently lead to a decline in conductivity [21,43]. Figure 5 present the energy-dispersive X-ray analysis (EDXA), and it was found that Zn was effectively embedded and dispersed into the PVA–CA–K_2_CO_3_ composites.

#### 3.1.4. Glass Transition Temperature of PVA–CA–K_2_CO_3_/ZnO-NPs

**Figure 6:**
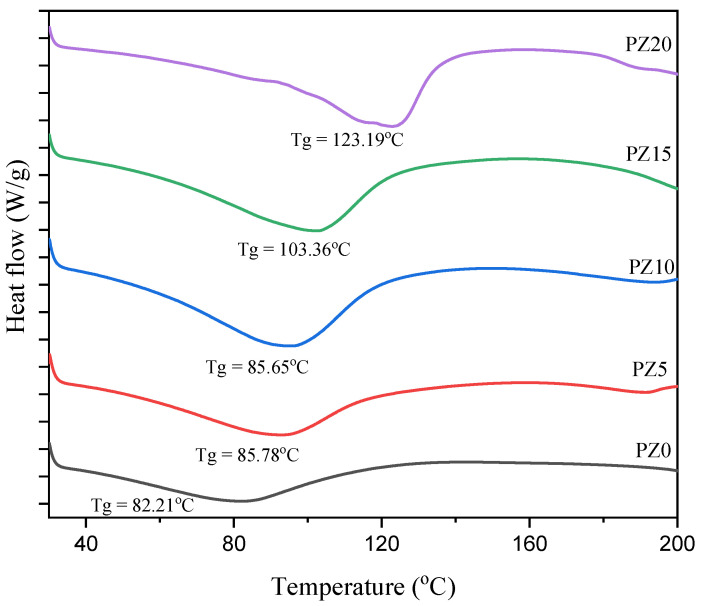
DSC scans for the developed composite polymer electrolyte.

To study the effect of the nanofiller on the polymer–salt electrolytes, DSC analysis was conducted on the samples. Figure 6 presents the DSC pattern of the polymer blend with no nanofiller and with varying concentrations in wt % of nanofillers. The lower value of glass transition temperature (*T_g_*) for all the samples evidently indicates the improved polymer flexibility and the promoted ion dynamics in terms of rapid ion transit across polymer chain coordination sites [34]. The *T_g_* peak for the blended polymer SPE with no nanofiller is observed to be located at 82.21 °C. On the other hand, with the successful incorporation of low wt % nanofiller (5–10 wt %), there was no significant shift in the *T_g_* peak toward a higher temperature, confirming the polymer–salt–nanofiller complex formation, which is ascribed to the coordination between the coordinate sites of the polymers and the Lewis base group and Lewis acid cation of the salt and the nanofiller [16,44].

This similarly indicates that ion mobility is improved by disrupting the polymer chain arrangement and creating disorder in the matrix, and this may promote the faster ion dynamics in the matrix that subsequently increase the conductivity of the electrolyte [45]. Low *T_g_* has been linked to a loss in crystallinity due to the addition of a filler substance. Electrolytes with a low *T_g_* are preferable because they allow the PVA–CA chains to be more plastic, allowing rapid ion transport. This ionic movement in polymer matrix systems could be linked to the limited fundamental relaxation classified by the *T_g_* of the electrolytes [46]. However, at high nanofiller content (15–20 wt %), it can be noticed that the peaks shift toward high temperature (103–123 °C), and this could be linked to the poor polymer–salt–nanofiller complexation due to the high amount of nanofiller, and this subsequently led to an increase in the crystalline region of the electrolytes, as observed earlier in the XRD results of Figure 2 [34,47,48]. Table 2 presents a summary of the *T_g_* and FWHM values for all samples with various concentrations of the filler. It can be observed that the two values (*T_g_* and FWHM) obtained are all in accordance with conductivities and potential windows achieved. 

### 3.2. Electrochemical Characterization

#### 3.2.1. SPE Resistance

**Figure 7:**
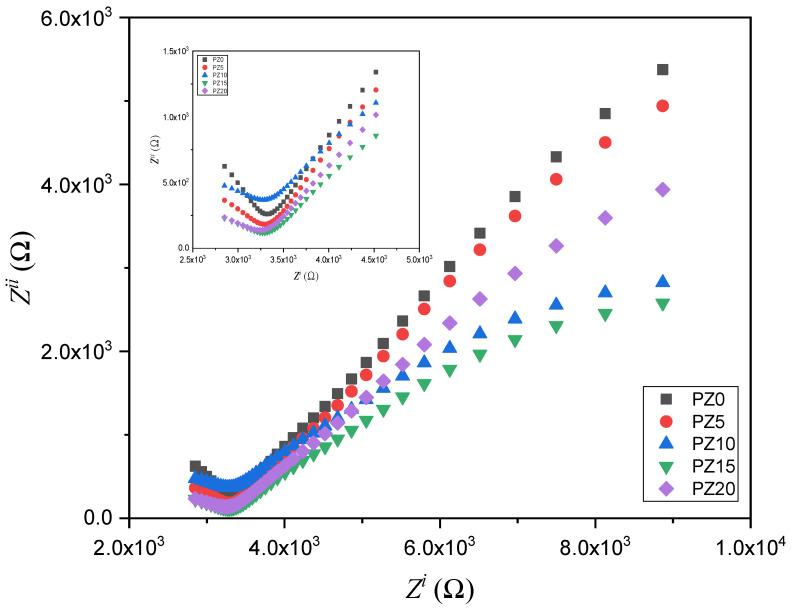
Cole–Cole plots for the PVA–CA–K_2_CO_3_/ZnO-NPs SPE composites at room temperature.

The EIS approach is used to analyze the conductivity of the SPEs and the electrode–electrolyte interface performance. The EIS results for all the prepared electrolytes with various concentrations of ZnO nanofiller are depicted in Figure 7. At a higher frequency range, the Nyquist plots for all the samples display a semicircle (inset in Figure 7), while spikes at a lower frequency signify the resistive and capacitive nature of the samples [49].

The semicircle shown in the high-frequency region indicates double-layer capacitance at the electrode–electrolyte interface owing to the ion migration, whereas the low-frequency spike was ascribed to the ion absorption, which indicates the capacitive nature of the electrolytes [50]. Nevertheless, it could be observed that the semicircle of the samples decreased with the successful incorporation of ZnO-NPs, which is attributed to a rise in the amorphous phase within the polymer, as well as due to the low T_g_ value, which leads to an increase in the electrochemical performance of the electrolytes [43,51]. The narrow semicircles with a tilted spike in the low- and high-frequency region in the Cole–Cole plots indicate that the samples contain mainly contain the resistive component [51]. The resistance is calculated from intercepts on the x-axis of the complex impedance plots. This high-frequency resistance is referred to as the bulk resistance (*R_b_*), and it reflects the bulk characteristics of the electrolyte from which the ionic conductivity (*σ*) is calculated using Equation (1) [12,36].

(1) $$\sigma =\frac{t}{R_{b}A}\;$$

where *A* is the electrode–electrolyte contact area (in cm^2^) and *t* is the thickness of the samples (in cm) [36,52].

The PZ0 with the larger semicircle has the highest bulk resistance, which was greatly lowered when ZnO nanofiller was added. The strong conducting and plasticizing properties of the ZnO-NPs play a key role in decreasing *R_b_* of the polymer blend. This demonstrates ZnO’s ability to enhance the interfacial interaction within the electrolyte and electrode. With this reduced bulk resistance, ionic mobility and transport could be improved, resulting in better ionic conductivity [53,54]. The *R_b_* decreases with increasing ZnO nanofiller concentration until it reaches 10 wt % and increases thereafter, indicating that the conductivity increased and subsequently decreased. This is due to the increased amount of mobile Zn^+^ cations within the polymer chains, which caused the chains to become more stretchy, and the amorphous region increased, resulting in increased ion migration [23].

#### 3.2.2. Ionic Conductivity

**Figure 8:**
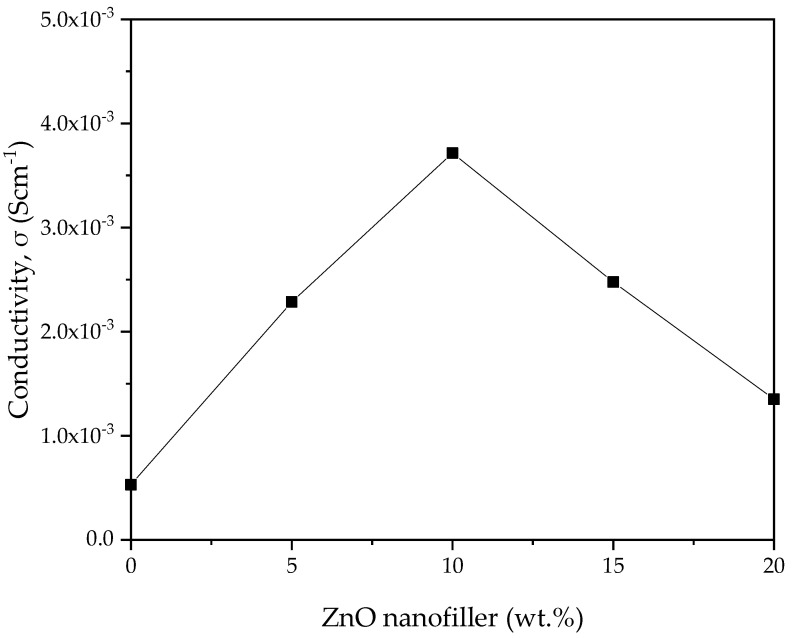
Ionic conductivity of PVA−CA−K_2_CO_3_/ZnO−NPs composite SPEs versus ZnO nanofiller concentration (in wt %) at room temperature.

The ionic conductivity (σ) versus ZnO-NP concentration in PVA–CA–K_2_CO_3_ composite SPEs obtained from the EIS spectra was computed using Equation (1), and the result is shown in Figure 8. The transportation of ions in the amorphous region is expected to be faster than in the crystalline region, and this could be linked to the poor arrangement of macromolecules in the amorphous region. The SPEs can produce faster ionic motion in the amorphous region that results in better conductivity for the electrolyte [51]. ZnO-NPs, as an important nanofiller, aid in flagging the coordinative bond between H and the weakly bonded -OH of the polymer. Consequently, the H atoms are facilitated to transfer from one position to another. As reported previously, the addition of filler such as ZnO to salted electrolyte material could alter the electrical characteristics in various ways. For instance, larger anion size is expected to dissociate and create a number of ions, thereby improving the conductivity through aiding the migration of ions in the polymer matrix by generating voids [55,56].

The bulk groups of cations assist in enhancing the conductivity through creating space for polymer segmental motion. Furthermore, the physicochemistry of ZnO nanofiller enhances the ionic conductivity. Consequently, the ion and polymer coordination might be disturbed, allowing conduction to continue [55]. The conductivity attained in this study is 3.70 × 10^–3^ Scm^–1^ at 10 wt % ZnO nanofiller (PZ0), which is much higher (almost by one order of magnitude) than the conductivity of 5.30 × 10^–4^ Scm^–1^ of the SPE without the ZnO nanofiller (PZ0) [31]. The conductivity then gradually decreased as the wt % of the ZnO nanofiller increased above 10 wt %. The substantial plasticizing effect of ZnO nanofiller is the reason for the increase in conductivity up to 10 wt % ZnO-NPs [41]. The strong influence of ZnO nanofiller helps to relax the strength of the polymer, which further enhances the elasticity of the polymer. The flexibility of the ZnO nanofiller enables the ions to easily migrate via the polymer matrix. Additionally, an increase in chain flexibility of the polymer was reported to improve polymer segmental motion and help ion movement within the composites [19]. ZnO-NPs are also known to affect the crystalline phase of the polymer that may degrade the temporary coordinative bond in the molecule within the region of crystallinity, which transforms the polymer chains into a flexible complex, as noticed in the XRD and FESEM in Figure 2 and Figure 4, respectively.

Nevertheless, the conductivity is observed to decrease beyond 10 wt % of ZnO-NPs, and this could be attributed to the buildup of ions and the accumulation of larger ions in the electrolytes. It was observed that a large number of ions in electrolytes leads ions to cluster, hindering the conducting pathways and averting the ions from migration, which results in a reduction in the moveable ions, and thus, a drop in conductivity is observed [57]. The increase in the number of ions owing to ion recombination can lead to the reduction in conductivity at higher wt % filler. Moreover, due to the excess number of ions in higher wt % ZnO-NPs, Zn^+^ may form ion pairs rather than offer ions for conduction [41]. 

The ionic conductivity attained is higher by about two orders of magnitude than that in previously reported studies using other polymers/salts [58]. The good performance of the SPEs with ZnO nanofiller in this study might be ascribed to the significant effect of the nanofiller, which assists in the movement of charge carriers [33]. Table 3 presents a comparison between blended SPEs prepared in this study with those of other studies published earlier. It is found that the electrolyte prepared in this study has a better conductivity than other polymer blends in previous studies incorporated with different fillers/plasticizers.

#### 3.2.3. Temperature versus Conductivity Relationship

**Figure 9:**
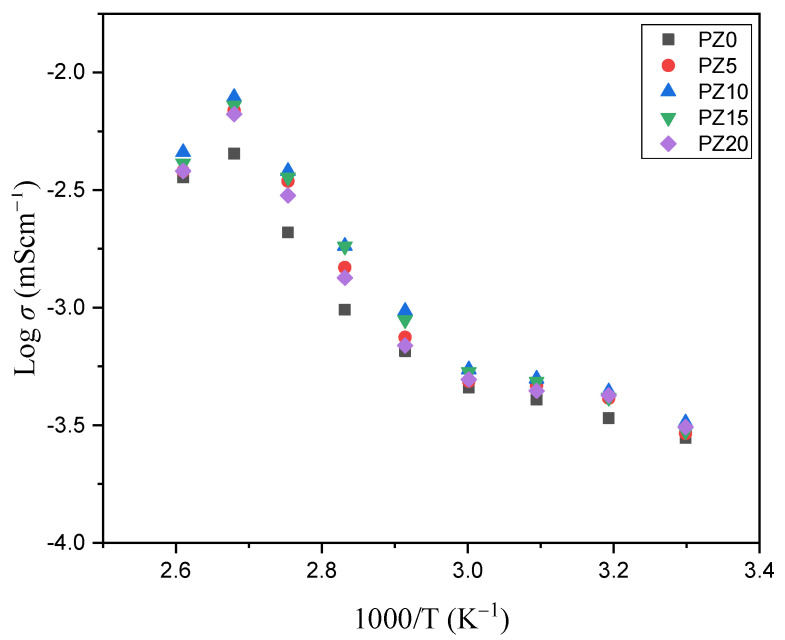
Conductivity versus temperature of PVA−CA−K_2_CO_3_/ZnO−NPs composite SPEs.

Figure 9 shows the conductivity versus temperature plot of the PVA–CA–K_2_CO_3_/ZnO-NPs composite SPEs, indicating a progressive increase in conductivity with the increase in temperature. The thermal alteration of the samples, particularly the polymer hosts, from semi-crystalline to more amorphous with an increase in ion migration and free Zn^+^ from ZnO nanofiller, is responsible for the rise in conductivity with the increase in temperature [30,61,62]. An earlier report similarly related the increase in conductivity of the electrolyte with the increase in temperature to the transformation of polymers from semi-crystalline to amorphous [63]. The linear difference in conductivity noticed at temperatures below 60 °C indicates that ion transport is facilitated by thermally induced mechanisms and thus follows the Arrhenius law. The Arrhenius model is a vital factor to study when dealing with the transportation of ions relative to temperature. The observed linear relationship points to a considerable phase transition in the prepared samples, signifying that the temperature dependence of the conductivity might indeed be designated by the Arrhenius model, as in Equation (2) [45]:

(2) $$\sigma =\sigma_{o}\exp\left[-\frac{E_{a}}{k_{B}T}\right]$$

where *E_a_* is the activation energy, *T* is the absolute temperature in Kelvin (K), and *σ_o_* denotes the pre-exponential factor.

Accordingly, an expansion of the polymers due to the polymer segmental motion at higher temperatures generates vacancies where the ions easily flow. Consequently, the mobility of ions improves and the ion cloud effect at the electrode–electrolyte interface is reduced. Equally, with the increase in temperature, the chain of the polymer grows a faster internal mode that causes segmental motion due to bond rotation. Therefore, the inter-chain ion hopping is preferred, which leads to the increase in conductivity with the temperature [64,65]. At temperatures above 373.15 K, nevertheless, the conductivity of the samples was shown to decrease. This reduction could be ascribed to the formation of elements inside the polymer matrix, which limits ion hopping and hence results in increased resistance in the majority of the samples [66,67].

#### 3.2.4. Activation Energy (*E_a_*)

**Figure 10:**
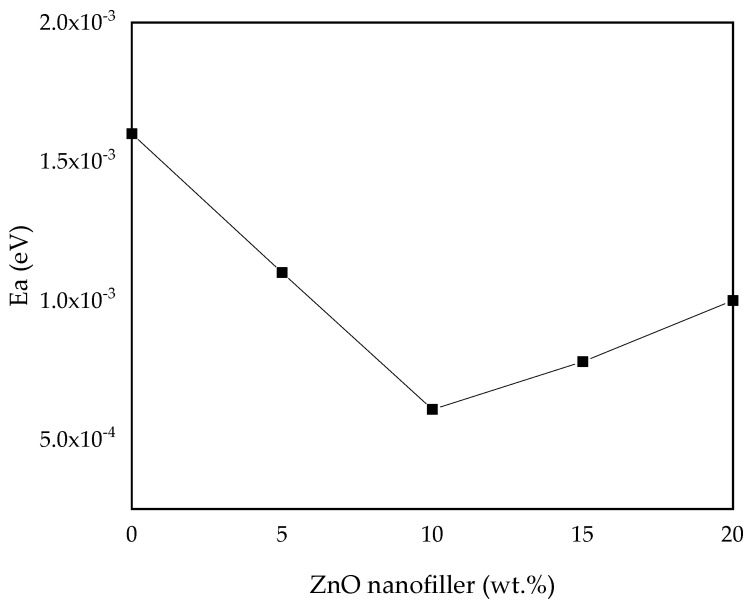
Activation energy (E_a_) versus ZnO-NP concentration.

To study the dynamic motion of ions in the prepared polymer electrolytes, the activation energy (*E_a_*) of all the samples was computed and analyzed by fitting it into the Arrhenius Equation (2) [30]. The results and values of *E_a_* of all the samples are shown in Figure 10 and Table 4, where the maximum conducting sample (PZ10) is found to have the lowest activation energy, which indicates that it is inversely proportional to the ionic conductivity. It shows that incorporation of ZnO nanofiller into the PVA–CA–K_2_CO_3_ composite tends to decrease the potential energy barriers for the ion migration, resulting in the decrease in activation energy [30]. Furthermore, the addition of fillers into the electrolyte was found to disturb the coordination within the backbone of the polymer and consequently needs less energy to break and form coordination bonds within the matrix of the polymer hosts. Thus, the diffusion of ions is promising, and this further enhanced the conductivity [16]. As reported previously, low *E_a_* is due to the tiny space within the transit sites delivered by the polymer blend. This result is similar to a large body of past research on various polymer electrolytes [13,30,46,66].

#### 3.2.5. Electrochemical Stability Window

**Figure 11:**
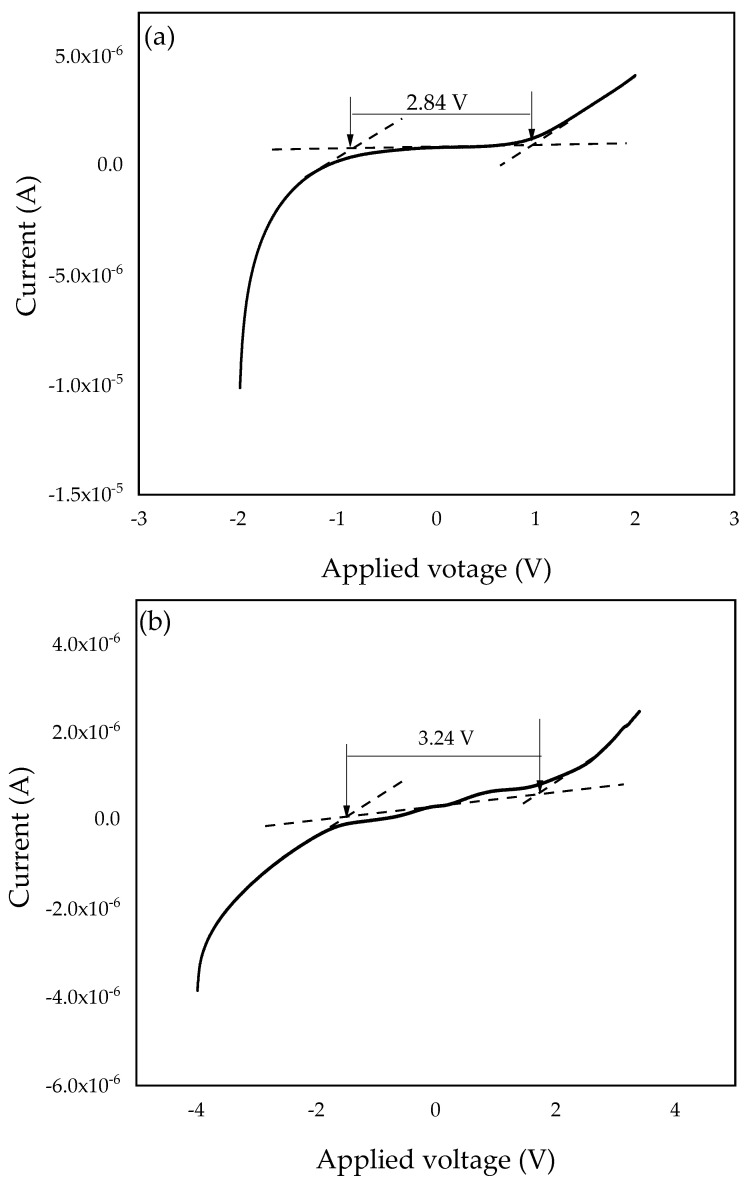
Linear sweep voltammetry (LSV) plots of the (**a**) PZ0 and (**b**) PZ10 composite SPE samples.

To investigate the electrochemical stability window of the SPEs, linear sweep voltammetry (LSV) was conducted. The working cell voltage range of SPEs is a significant feature to investigate since it demonstrates the electrolyte’s capacity to maintain the operating voltage of electrochemical devices [67]. Therefore, the electrochemical stability window of the PZ0 and the optimized PVA–CA–K_2_CO_3_/ZnO-NPs composite (PZ10) were investigated and studied at a 5 mVs^–1^ scan rate, and the resultant voltammograms are presented in Figure 11a,b. For the PZ0 sample, we found that the current remained constant while the voltage increased until the sample reached a maximum (V_max_) [68]. Sample PZ10 in this study was determined to have a wide electrochemical stability window of 2.84 V, which can be linked to the salt particles. The strong effects of K_2_CO_3_ and the dielectric constant of the polymers can impact electrochemical stability, resulting in a higher charge carrier concentration [69].

However, the stabilities of the SPEs increased with the incorporation of ZnO nanofiller into the polymer membrane, and the electrochemical stability window voltage further improved to 3.24 V, as shown in Figure 11b, with the incorporation of 10 wt % ZnO nanofiller. The observed increase in voltage stability could be attributed to ZnO functioning as a filler that binds to the polymer–salt chain and causes the electrolyte to not decompose rapidly. Similar findings have previously been published, with the authors claiming that fillers can help expand an electrolyte’s stability window [16,27]. The increase in the electrochemical stability window with the incorporation of ZnO-NPs as a nanofiller may be due to the coordination within the polymers, K_2_CO_3_, and ZnO-NPs that changed the physical properties of the polymer and consequently enhanced the electrochemical stability [27,70]. The result attained in this study using ZnO-NPs as a nanofiller is much higher than the conventional recommended value for electrochemical stability (1.7 V) of an electrolyte for application in any energy storage devices and higher than other studies reported earlier using different fillers [27,71]. Hence, from the results obtained in this study, the PVA–CA–K_2_CO_3_/ZnO-NPs SPE composites has potential to be used as an electrolyte in an all-solid-state for energy storage devices.

### 3.3. Transference Number (TNM) Measurements

To further support the conductivity study of the prepared samples, the analysis of the ion transference number (t_ion_) and electron transference number (t_el_) is vital. The transference number is defined as the number of moles of ion transferred for one Farad of charge transferred. Preferably, the transference number, *t*^+^, should be close to 1 in a high-conductivity polymer electrolyte, and is calculated as in Equation (3) [72]:

(3) $$t^{+}=\frac{I_{s}}{I_{o}}\;$$

where *I_o_* and *I_s_* denote the initial and steady-state cell current, respectively.

**Figure 12:**
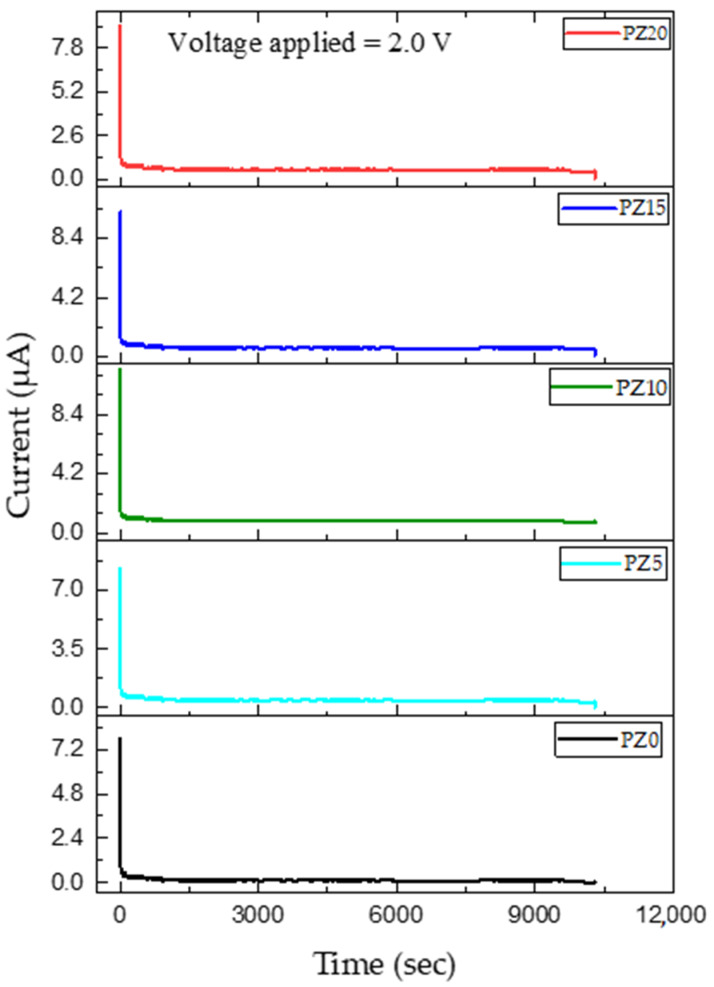
Polarization curve of ion transference number as a function of time of prepared SPEs.

In this study, the transference number equivalent to ionic transport (t_i_) was evaluated in PVA–CA–K_2_CO_3_/ZnO-NPs SPE systems using Wagner’s polarization technique [72]. Prior to the analysis, the sample was sandwiched between two stainless steel blocking electrodes and a fixed voltage was applied, and the current is studied as a function of time until the saturation limit is reached. The resultant plot of the transference number as a function of time for the samples is shown in Figure 12. It can be observed that the initial current of all the samples is high and this could be linked to the flow of electrons and ions, and the current thereafter decreased with an increase in time.

Previous research has reported that the initial decrease in the current is due to the polarization effects, resulting in ion buildup at the electrolyte–electrode interface. Similarly, the remaining current is due to electrons as ions become blocked with time [73]. The ion transference number (t_ion_) and electron transference number (t_el_) achieved in this study for all the samples are summarized in Table 5, and all the results were found to be in accordance with the values of ionic conductivity and LSV achieved. The suitable result of the transference number achieved is due to the fast ion migration in the polymer dispersed with ZnO nanofillers. It was found that the cation from the salt/plasticizer coordinates with the electron-rich group of the polymer and thus modifies the chain arrangement of the polymer, which results in the increment in the amorphous content of the electrolyte. This increment in the amorphous content expedites the faster movement of ions that led to improved ion TNM [74]. Hence, this indicates that the charge transport in this electrolyte is mainly due to charge carriers. The good transference number could be ascribed to the influence of ion–ion and polymer–ion interactions on the macroscopic transport parameters. The ion transference number attained in this study is adequate for application in any energy storage device [72,73].

## 4. Conclusions

Free-standing polymer electrolyte films (SPEs) based on PVA blended with reinforced cellulose acetate (PVA–CA) and K_2_CO_3_ composites incorporated with different contents of ZnO nanofiller (5–20 wt %) were synthesized and characterized. The morphology of the samples indicated that the ZnO nanofiller was regularly dispersed at lower amounts and started to agglomerate at higher wt %. The structural characterization based on XRD and FTIR revealed that the polymers, the salt, and the nanofiller are compatible and blended by means of interfacial adhesion and coordination via hydrogen bonding due to the existence of –OH of the polymer blend. This indicates the occurrence of good coordination between the PVA–CA–K_2_CO_3_ and ZnO nanofiller that leads to a favorable distribution of ions within the polymers. The highest electrochemical performance was achieved by 10 wt % ZnO-NPs with an optimum ionic conductivity (at room temperature), high potential window, and the lowest activation energy (*E_a_*) of 3.70 × 10^–3^ Scm^−1^, 3.24 V, and 6.08 × 10^–4^ eV, respectively. The favorable performance observed was due to the increase in the number of charge carriers and the effect of electrode polarization owing to the strong effect of ZnO nanofiller. Temperature and frequency dependence were found to approximately follow the Arrhenius model, at least below 60 °C, which established that the electrolytes in this study were thermally activated below this temperature. Therefore, the results achieved in this study confirm that polymer blend SPEs based on PVA–CA–K_2_CO_3_ with ZnO nanofiller have considerable potential for application in portable electrochemical devices.

## Figures and Tables

**Table 1:** Variations of ZnO-NPs in the PVA–CA–K_2_CO_3_/ZnO SPE composites.

Description	PVA–CA Polymer Blend Ratio	K_2_CO_3_ Content (wt %)	ZnO-NPs Content (wt %)
PZ0	80:20	20	0
PZ5	80:20	20	5
PZ10	80:20	20	10
PZ15	80:20	20	15
PZ20	80:20	20	20

**Table 2:** Summary of *T_g_* and FWHM of polymer blend SPEs.

Samples	*T_g_* (°C)	FWHM
PZ0	82.21	0.642
PZ5	85.78	0.470
PZ10	85.65	0.405
PZ15	103.36	0.367
PZ20	123.19	0.288

**Table 3:** Comparison of the conductivities at room temperature of polymer blend SPEs from the literature.

Polymers	Salt	Filler/Plasticizer	Conductivity (S/cm)	Ref.
PVA–CA	K_2_CO_3_	ZnO-NPs	3.70 × 10^–3^	This study
Starch–chitosan	NH_4_I	Glycerol	1.28 × 10^–3^	[58]
Chitosan–PEO	NH_4_NO_3_	Ethylene carbonate (EC)	2.06 × 10^–3^	[56]
Chitosan-based solid biopolymer	NH_4_Br	Glycerol	1.51 × 10^–3^	[59]
Pectin–methylcellulose	K_3_PO_4_	Glycerol	3.00 × 10^–4^	[24]
PEO–MC	NH_4_I	Polyethylene glycol	3.37 × 10^−3^	[10]
PVA	NH_4_NO_3_	ZnO-NPs	4.71 × 10^–4^	[30]
PEO	LiClO_4_	ZnO	1⋅28 × 10^–5^	[60]

**Table 4:** Activation energy (*E_a_*) of the prepared SPEs based on PVA–CA–K_2_CO_3_/ZnO-NPs.

S/N	Samples	Activation (*E_a_*) (eV)
1	PZ0	1.60 × 10^–3^
2	PZ5	1.00 × 10^–3^
3	PZ10	6.08 × 10^–4^
4	PZ15	7.80 × 10^–4^
5	PZ20	1.10 × 10^–3^

**Table 5:** Transference number of the PVA–CA–K_2_CO_3_/ZnO-NPs composite SPEs.

Samples	Electron Transference Number (t_el_)	Transference Number (t_ion_)
PZ0	0.090	0.909
PZ5	0.089	0.911
PZ10	0.034	0.965
PZ15	0.078	0.922
PZ20	0.082	0.918

## Data Availability

The data presented in this study are available on request from the corresponding author. The data are not publicly available due to privacy.
